# EXTERNAL PRINGLE MANEUVER IN LAPAROSCOPIC LIVER RESECTION: A SAFE, CHEAP AND REPRODUCIBLE WAY TO PERFORM IT

**DOI:** 10.1590/0102-672020200004e1555

**Published:** 2021-01-25

**Authors:** Klaus STEINBRÜCK, Reinaldo FERNANDES, Marcelo D’OLIVEIRA, Rafaela CAPELLI, Renato CANO, Hanna VASCONCELOS, Luiza BASILIO, Marcelo ENNE

**Affiliations:** 1 Hepatobiliary Multidisciplinary Group, Rio de Janeiro, RJ, Brazil; 2 Bonsucesso Federal Hospital - Health Ministry, Hepatobiliary Surgery, Rio de Janeiro, RJ, Brazil; 3 Ipanema Federal Hospital - Health Ministry, Hepatobiliary Surgery, Rio de Janeiro, RJ, Brazil

**Keywords:** Liver, Laparoscopy, Hepatectomy, Hemorrhage, Fígado, Laparoscopia, Hepatectomia, Hemorragia

## Abstract

**Background:**

Laparoscopic liver resection is performed worldwide. Hemorrhage is a major complication and bleeding control during hepatotomy is an important concern. Pringle maneuver remains the standard inflow occlusion technique.

**Aim:**

Describe an extracorporeal, efficient, fast, cheap and reproducible way to execute the Pringle maneuver in laparoscopic surgery, using a chest tube.

**Methods:**

From January 2014 to March 2020, our team performed 398 hepatectomies, 63 by laparoscopy. We systematically encircle the hepatoduodenal ligament and prepare a tourniquet to perform Pringle maneuver. In laparoscopy, we use a 24 Fr chest tube, which is inserted in the abdominal cavity through a small incision. We thread the cotton tape through the tube, pulling it out through the external end, outside the abdomen. To perform the tourniquet, we just need to push the tube as we hold the tape, clamping both with one forceps.

**Results:**

The 24 Fr chest tube is firm and works perfectly to occlude blood inflow as the cotton band is tightened. It has an internal diameter of 5,5 mm, sufficient for a laparoscopic grasper pass through it to catch the cotton band, and an external diameter of 8 mm, which allows to be inserted in the abdomen through a tiny incision. The cost of this tube and the cotton band is less than US$ 1. No complications related to the method were identified in our patients.

**Conclusions:**

The extracorporeal Pringle maneuver presented here is a safe, cheap and reproducible method, that can be used for bleeding control in laparoscopic liver surgery.

## Graphical abstract

[Fig f1a]

**Figure f1a:**
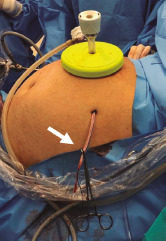
Tourniquet for right hepatectomy tightened and secured with forceps (arrow).

[Fig f1as]

**Figure f1as:** Torniquete para hepatectomia direita apertado e preso com uma pinça (seta).

## RESUMO

**Racional:**

Ressecções hepáticas laparoscópicas são realizadas em todo mundo. A hemorragia é complicação grave e o controle do sangramento durante a hepatotomia é preocupação importante. A manobra de Pringle continua sendo a técnica padrão de oclusão do influxo sanguíneo.

**Objetivo:**

Descrever uma maneira eficiente, rápida, barata e reproduzível de executar a manobra de Pringle extracorpórea, em operação laparoscópica, utilizando um dreno de tórax.

**Métodos:**

De janeiro/2014 a março/2020, realizamos 398 hepatectomias, 63 por laparoscopia. Nós sistematicamente laçamos o ligamento hepatoduodenal e preparamos um torniquete para a manobra de Pringle. Na laparoscopia, usamos um dreno de tórax 24 Fr, inserido na cavidade abdominal através de uma pequena incisão. Passamos a fita de algodão através do tubo, puxando-a pela extremidade externa, fora do abdome. Para apertar o torniquete, basta pressionar o tubo enquanto seguramos a fita, prendendo ambos com uma pinça.

**Resultados:**

O dreno de tórax 24 Fr é firme e funciona perfeitamente para ocluir influxo de sangue, à medida que apertamos o torniquete. Tem diâmetro interno de 5,5 mm, suficiente para passar uma pinça laparoscópica e puxar a fita de algodão, e um diâmetro externo de 8 mm, permitindo a inserção no abdome através de uma pequena incisão. O custo do tubo e fita é inferior a US$ 1, valor insignificante. Não foram identificadas complicações relacionadas ao método em nossos pacientes.

**Conclusões:**

A manobra extracorpórea de Pringle apresentada aqui é método seguro, barato e reproduzível, que pode ser utilizado para o controle do sangramento em hepatectomias laparoscópicas.

## INTRODUCTION

Laparoscopic liver resection (LLR) is a reality and is performed worldwide for almost all types of liver surgeries, from edge resections to adult living donors^2^ ^,^ ^9^. Hemorrhage is still a major complication and bleeding control during hepatic transection is an important concern. Pringle maneuver (PM) remains the standard inflow occlusion technique and herein we describe an extracorporeal, efficient, fast, cheap and reproducible way to execute it using a chest tube.

From January 2014 to March 2020, our team performed 398 hepatectomies, 63 by laparoscopy. For LLR, we systematically encircle the hepatoduodenal ligament and prepare a tourniquet to perform PM whenever it is necessary. Initially, we use to do it intra-corporeally, but we modified our technique to extra-corporeal tourniquet as we noticed it was easier and faster to be tightened.

## METHOD

This work was approved by institutional ethics committee with number 089/2019.

### Technique

**FIGURE 1:**
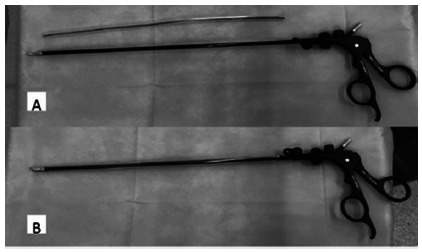
A) 24 Fr chest tube and laparoscopic grasper; B) grasper inside the tube, ready to be inserted into the abdomen.

**FIGURE 2:**
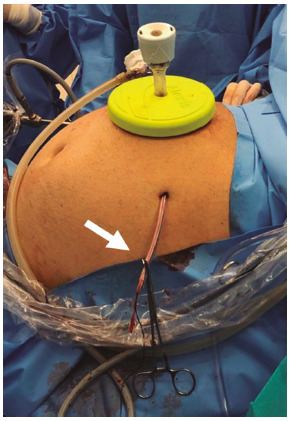
Tourniquet inserted through the left flank for right hepatectomy; the tourniquet is tightened and clamped with a forceps (arrow); the 24 Fr chest tube doesn’t bend while subjected to pressure.

Once the pneumoperitoneum is completed and the hepatoduodenal ligament has been exposed, the pars flaccida of the gastrohepatic ligament is opened and a 10 mm “gold finger” articulated dissector is passed behind the hepatoduodenal ligament - this movement is easier to be done from the right side of the ligament to the left side - to encircle it with a cotton tape. The tape is then pulled to the halfway point and both ends are held together with a laparoscopic grasper. To make the tourniquet, we use a 24 Fr chest tube, which is inserted in the abdominal cavity through a 1 cm incision, with a laparoscopic grasper inside it, to maintain it straight (Figure 1). Once the tube is introduced into the abdomen, we use the same grasper inside the tube to catch both ends of the cotton tape and thread it through the internal end of the tube, pulling it out through the external end, outside the abdomen. The side of the abdominal wall that we place the tourniquet depends on the type of liver resection: for right side resections, we place it in the left flank, and for left side resections, in the right flank. To perform the tourniquet maneuver, we just need to push the tube as we hold the tape, clamping both with one forceps (Figure 2).

## RESULTS

After changing our technique from intracorporeal to extracorporeal tourniquet, we performed 35 laparoscopic hepatectomies. We had success to prepare the extra-corporeal tourniquet, as described before, in all of those. No complications related to the method were identified in our patients.

## DISCUSSION

Laparoscopic liver surgery should always be performed with the patient’s safety in mind, as in open surgery. Taking this into account, unnecessary bleeding during liver transection is a major concern. Inflow occlusion by Pringle maneuver is an established method to decrease hemorrhage during hepatectomy and is widely used^3^. Many methods have been described to perform PM in LLS^1^ ^,^ ^5^ ^,^ ^6^, but the tourniquet method seems to be the easier and safer. The grate advantage of the tourniquet PM is that it can be prepared before liver transection and, once the hepatoduodenal ligament is taped, the tourniquet can be tightened quickly, reducing blood lost in case of an unexpected vascular lesion. The same process could be really difficult with a clamp or laparoscopic bulldog, especially in a blood covered field, with the risk of damaging structures of the porta hepatis.

When we started our program of LLS, we opted to do the PM intra-corporeally, as described by Cherqui et al.^4^, but it was sometimes difficult to be tightened and took some important minutes to be achieved, especially during bleeding. After reading the papers from Rotellar et al.^8^ and Patriti et al.^7^, we modified our technique to extra-corporeal tourniquet. We noticed that this way was easier to be tightened and could be performed by the auxiliary surgeon, avoiding distraction from the bleeding site.

The tube used in the extra-corporeal tourniquet needs to be rigid enough not to bend when subjected to pressure. We prefer to use a 24-Fr chest tube, which can be easily find in any hospital. This kind of tube is firm and works perfectly to occlude blood inflow as the cotton band is tightened. It has an internal diameter of 5,5 mm, sufficient for a laparoscopic grasper pass through it to catch the cotton band, and an external diameter of 8 mm, which allows to be inserted in the abdomen through a tiny incision. The cost of this tube and the cotton band is less than US$ 1, a negligible value, considering the total cost of laparoscopic surgery.

In our series, we had no complications related to the placement or during the use of this type of extra-corporeal tourniquet. Probably, extensive adhesions to the hepatoduodenal ligament and previous liver surgeries could make it difficult to perform this type of laparoscopic PM.

## CONCLUSION

The extracorporeal Pringle maneuver presented here is a safe, cheap and reproducible method, which can be used for bleeding control in laparoscopic liver surgery.
